# Biosensors for Studying Neuronal Proteostasis

**DOI:** 10.3389/fnmol.2022.829365

**Published:** 2022-03-08

**Authors:** Irina Dudanova

**Affiliations:** Molecular Neurodegeneration Group, Max Planck Institute of Neurobiology, Martinsried, Germany

**Keywords:** proteostasis, protein quality control, protein folding, neuron, biosensor, protein misfolding diseases

## Abstract

Cellular health depends on the integrity and functionality of the proteome. Each cell is equipped with a protein quality control machinery that maintains protein homeostasis (proteostasis) by helping proteins adopt and keep their native structure, and ensuring the degradation of damaged proteins. Postmitotic cells such as neurons are especially vulnerable to disturbances of proteostasis. Defects of protein quality control occur in aging and have been linked to several disorders, including neurodegenerative diseases. However, the exact nature and time course of such disturbances in the context of brain diseases remain poorly understood. Sensors that allow visualization and quantitative analysis of proteostasis capacity in neurons are essential for gaining a better understanding of disease mechanisms and for testing potential therapies. Here, I provide an overview of available biosensors for assessing the functionality of the neuronal proteostasis network, point out the advantages and limitations of different sensors, and outline their potential for biological discoveries and translational applications.

## Introduction

**Figure 1:**
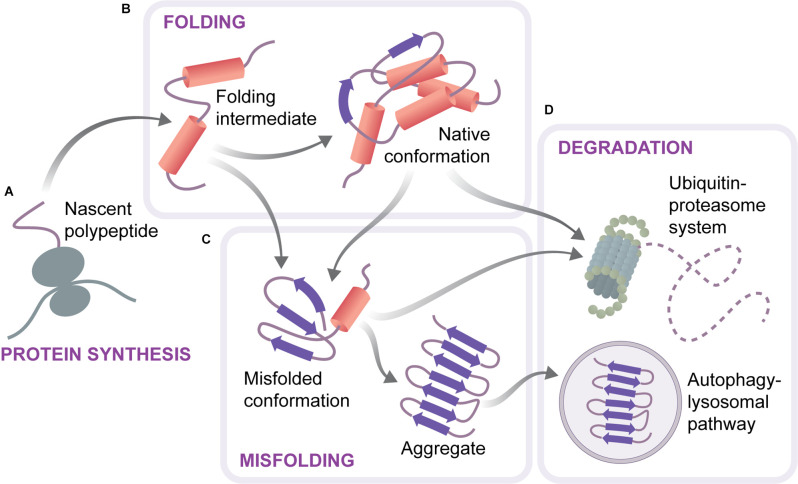
Scheme of the proteostasis system. Proteins are synthesized on ribosomes as unfolded polypeptides **(A)**. They reach their native conformation through a folding process that is assisted by chaperones and can include several folding intermediates **(B)**. Proteins can also adopt alternative, misfolded conformations, which are prone to aggregation and lead to the formation of amyloid-like aggregates **(C)**. Aberrant or excessive proteins are removed by two cellular degradation systems, the ubiquitine-proteasome system and autophagy **(D)**.

To maintain cellular health, proteins have to be synthesized in required amounts, correctly folded and assembled into complexes, and turned over at appropriate rates. Cells possess an elaborate protein quality control machinery for accomplishing these tasks and guarding protein homeostasis (proteostasis; Labbadia and Morimoto, 2015; Hipp et al., 2019). This machinery, referred to as the proteostasis network, includes protein synthesis components, molecular chaperones, the ubiquitin-proteasome system (UPS) as well as the autophagy-lysosomal system (Figure 1). Chaperones guide proteins on their folding pathways, keep them in the proper conformation, and ensure the timely removal of excess or damaged proteins by degradation systems (Shiber and Ravid, 2014; Balchin et al., 2016). Monomeric proteins are degraded by the UPS upon their unfolding, while large multimeric complexes are cleared through the autophagy-lysosomal pathway (Dikic, 2017). Mutations, mRNA processing defects, translation errors, and various types of external stress can lead to protein misfolding, imposing a burden on the proteostasis network (Hipp et al., 2019). Misfolded species are normally recognized by chaperones and either refolded or targeted for degradation. However, if not efficiently cleared, misfolded proteins might accumulate and form aggregates (Figure 1), with potentially harmful consequences for the cells. Indeed, protein aggregates are a common feature of protein misfolding diseases, including neurodegenerative disorders such as Alzheimer’s, Parkinson’s, or Huntington’s disease. As proteostasis capacity undergoes an age-related decline, aging represents a common risk factor for these diseases (Douglas and Dillin, 2010; Brehme et al., 2014; Vilchez et al., 2014; Hipp et al., 2019). Moreover, mutations in components of the proteostasis network are often associated with neurodegeneration (Labbadia and Morimoto, 2015).

The composition and function of the proteostasis network, as well as susceptibility to its disturbances, varies between tissues and cell types (Guisbert et al., 2013; Labbadia and Morimoto, 2015). Neurons are particularly vulnerable to protein misfolding for several reasons. As postmitotic cells, they are unable to rejuvenate and redistribute damaged protein species through cell divisions, or clear them as efficiently as has been shown for neural stem cells (Vilchez et al., 2012; Bufalino et al., 2013; Moore et al., 2015; Leeman et al., 2018). In addition, neurons are exceptionally long-lived and have to endure proteotoxic insults that accumulate during the whole life span. Interestingly, at least some types of neurons also fail to efficiently activate the heat shock response (Marcuccilli et al., 1996; Batulan et al., 2003), a canonical stress response that leads to upregulation of chaperones and alleviates proteotoxic stress (Morimoto, 2011). Finally, proteostasis capacity can vary considerably between neuronal cell types, depending on their repertoire of proteostasis network components and regulators (Tagawa et al., 2007; Tsvetkov et al., 2013).

The proteostasis system is amenable to pharmacological manipulation, and interventions that enhance the cellular proteostasis capacity hold great promise for the treatment of neurodegeneration (Balch et al., 2008; Labbadia and Morimoto, 2015; Baranczak and Kelly, 2016; Hommen et al., 2021). For example, treatment with the small molecule arimoclomol, which improves proteostasis by potentiating the heat-shock response and inducing expression of several chaperones, ameliorated disease signs in mouse models of several neurodegenerative proteinopathies (Kieran et al., 2004; Malik et al., 2013; Ahmed et al., 2021), and also produced promising results in amyotrophic lateral sclerosis patients (Benatar et al., 2018). A prerequisite for the development of such therapeutic strategies for different neurodegenerative conditions is a thorough understanding of the changes in neuronal proteostasis in healthy aging and during the course of the disease. In addition, the success of potential proteostasis-targeting interventions has to be monitored with a robust readout. To this end, there is a need for proteostasis biosensors that can measure the functionality of neuronal protein quality control *in vivo*. Several such sensors have been developed and used in different model organisms. In the following sections, I will review the major classes of proteostasis sensors, their advantages and limitations, and their applications in neurons.

## Proteostasis Biosensors

Biosensors are tools that specifically detect certain biomolecules and provide information on their concentration, localization, and/or function. Biosensors have proven extremely useful in biomedical research by allowing visualization and quantification of dynamic processes in living cells (Velasco-Garcia, 2009; Greenwald et al., 2018). In the field of neurosciences, specialized sensors have been developed e.g., for interrogating synaptic function, monitoring intracellular trafficking, and detecting activation of various signaling cascades (Choquet et al., 2021; Laviv and Yasuda, 2021). Most commonly, such sensors rely on fluorescence, but some use other readouts such as luminescence.

Proteostasis sensors are used to monitor the functional state of the protein quality control system in living cells and whole organisms. Their major current and future applications include mechanistic studies of proteostasis pathways in health and disease, development of proteostasis-modifying drugs, as well as diagnostics and treatment monitoring of protein misfolding disorders.

Various reporters exist for the proteostasis network on the whole, as well as for some of its parts. Here, I will focus on general proteostasis sensors that report primarily on the folding capacity of cells, with a particular emphasis on the tools suitable for investigating neuronal proteostasis in animal models. These sensors typically either detect endogenous proteins in an unfolded state or are themselves chaperone clients whose unfolded state triggers changes in their fluorescence, cellular distribution and/or function. In addition to folding sensors, specialized tools have been developed for probing the UPS system and autophagy (reviewed in Lindsten et al., 2003; Matilainen et al., 2016; Klionsky et al., 2021). Finally, sensors for monitoring certain chaperones and canonical stress responses such as the heat shock response or the unfolded protein response of the endoplasmic reticulum are also available (e.g., Batulan et al., 2003; Morley and Morimoto, 2004; van Oosten-Hawle et al., 2013; Kijima et al., 2018; Pereira et al., 2018; Miles and van Oosten-Hawle, 2020; Shen et al., 2021), but will not be discussed in detail here.

## Types of Proteostasis Sensors

General proteostasis sensors can be divided into three major groups: small molecule sensors, genetically encoded sensors based on endogenous proteins, and genetically encoded sensors based on ectopic proteins (Table 1).

**Table 1:** Available proteostasis sensors.

Sensor type	Main advantages (+) and limitations (−)	Examples of sensors	Readout(s) of proteostasis impairments	Model systems the sensor has been used in	References
Small molecules	(+) Precise temporal control of application, no burden on the protein quality control system, ideal for large screens	TPE-MI, TPE-NMI	Turn-on fluorescence	Cell lines, iPSC-derived neural precursors	Chen et al. (2017) and Zhang et al. (2019)
	(−) No cell type specificity, not suitable for long-term *in vivo* studies	NTPAN-MI	Turn-on fluorescence with polarity-sensitive spectrum	Cell lines	Owyong et al. (2020)
		VB1Cl	Turn-on fluorescence	Cell lines	Mu et al. (2021)
Endogenous proteins	(+) Suitable for long-term *in vivo* studies (−) Burden on the protein quality control system, potential loss-of-function effects	Paramyosin(ts), dynamin(ts), perlecan(ts), unc-45(ts), ras(ts), gas-1(ts), acetylcholine receptor(ts)	Different readouts depending on the specific protein, e.g., at cellular level: mislocalization of the sensor, altered protease sensitivity; at organismal level: embryonic lethality/ development arrest, movement impairment, egg-laying defect	*C. elegans*	Gidalevitz et al. (2006) and Ben-Zvi et al. (2009)
Ectopic proteins	(+) Suitable for long-term *in vivo* studies, no loss-of-function effects	Fluc-EGFP	Decrease in bioluminescence, formation of Fluc-EGFP foci	Cell lines, primary neurons, *C. elegans*, transgenic mice	Gupta et al. (2011) and Blumenstock et al. (2021)
	(−) Burden on the protein quality control system	AgHalo	Turn-on fluorescence	Cell lines	Liu et al. (2017), Fares et al. (2018), and Liu et al. (2018)
		Retroaldolase	Formation of fluorescent aggregates	Cell lines	Liu et al. (2015)
		Barnase FRET sensor	FRET	Cell lines	Wood et al. (2018)

### Small Molecule Sensors

**Figure 2:**
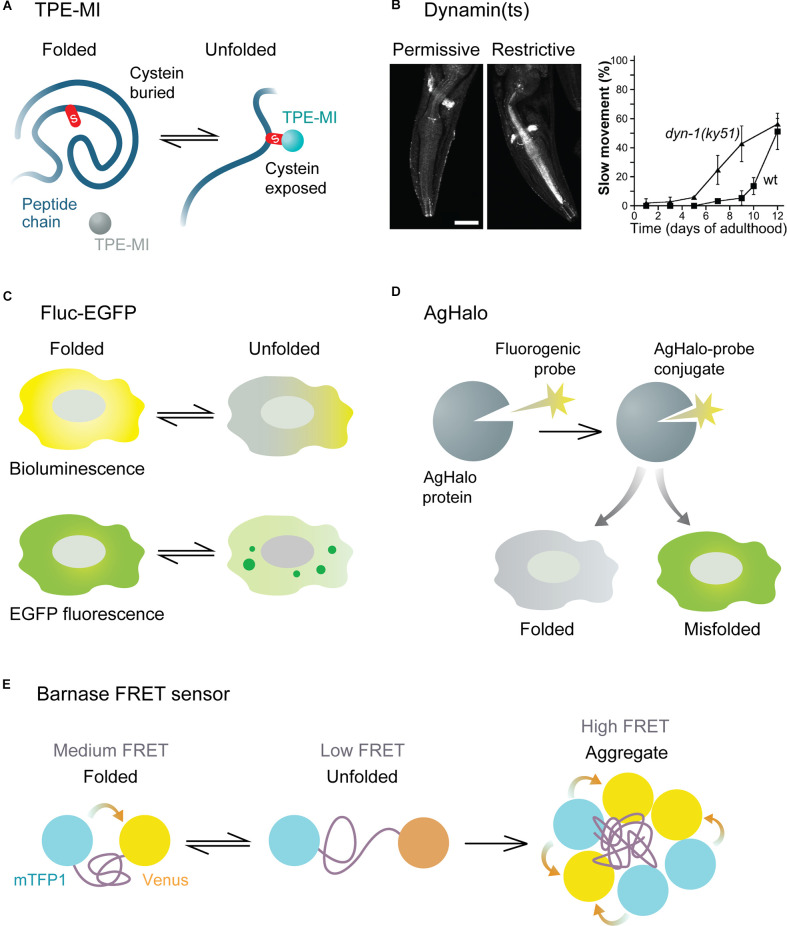
Functional principles of various proteostasis sensors. **(A)** TPE-MI small molecule sensor becomes fluorescent upon binding to free cysteine residues of unfolded proteins. **(B)** Dynamin temperature sensitive (ts) mutant mislocalizes (left) and causes movement defects (right) in conditions of impaired proteostasis. Images are adapted with permission from Ben-Zvi et al. (2009). **(C)** Fluc-EGFP sensor displays reduced luciferase activity and forms fluorescent foci when not folded correctly. **(D)** AgHalo sensor forms a conjugate with a small molecule probe, which emits fluorescence when the sensor is misfolded. **(E)** Barnase sensor displays different levels of FRET in folded, unfolded, and aggregated states.

Small molecule sensors are represented by fluorogenic small molecules that become fluorescent upon binding to free thiol groups of cysteine residues in unfolded proteins. Free cysteines not engaged in disulfide bonds are usually buried within the three-dimensional structure of native proteins but are exposed in unfolded ones. These sensors, therefore, allow estimating the pool of unfolded proteins in a cell. An example of such a sensor is tetraphenylethene maleimide (TPE-MI, Figure 2A). In addition to immunofluorescence and flow cytometry applications, TPE-MI is well suited for proteomics approaches, as its binding leads to a change in cysteine residue-containing peptides, and allows identifying the proteins that are unfolded under certain conditions (Chen et al., 2017). The drawbacks of TPE-MI are its low water solubility and absorption peak in the ultraviolet range. Its optimized derivative, TPE-NMI, is more hydrophilic and shows a red-shifted spectrum, making it better suited for commonly used lasers (Zhang et al., 2019). Importantly, the TPE-MI and TPE-NMI sensors have been verified in neuron-like Neuro2A cells, as well as in iPSC-derived primitive neural precursor cells, in both cases revealing reduced folding capacity in the presence of mutant Huntingtin prior to the formation of visible aggregates (Chen et al., 2017; Zhang et al., 2019). An interesting recent addition to the suite of maleimide sensors is NTPAN-MI, described as a “molecular chameleon” (Owyong et al., 2020). Due to its polarity-sensitive emission profile, it can visualize the subcellular changes in polarity that occur in the local environment of unfolded proteins. As different subcellular compartments display inherent differences in polarity and its dynamics upon proteotoxic stress, NTPAN-MI can provide a refined spatial map of proteostasis disbalance in a cell. This would be particularly interesting to investigate in morphologically complex cells like neurons.

One of the limitations of maleimides is their reduced stability in certain pH conditions (Koniev and Wagner, 2015). This shortcoming was addressed by the development of another thiol-reactive small molecule, the BODIPY-based probe VB1Cl, which is stable in a broad pH range (Mu et al., 2021).

Small molecule sensors do not impose an additional burden on the protein quality control system, are easy to use, and allow precise temporal control of the experiments, as they are membrane-permeable and can be bath-applied to cultured cells. Moreover, they provide a direct measure of the state of the cellular proteome due to their binding to endogenous unfolded proteins. Assays using these sensors are easily scalable and ideal for large screens, e.g., for testing proteostasis-correcting molecules in iPSC-derived neurons. However, small molecule sensors cannot be specifically targeted to certain tissues or cell types, and are not suitable for long-term studies over the lifetime of an organism. While well established in cell culture settings, they have not yet been tested in animal models. Further potential improvements in these sensors include the development of molecules with fluorescence in different parts of the spectrum, for easier combination with other available fluorescent tools and imaging techniques.

### Genetically Encoded Sensors

Genetically encoded reporters are ideal for longitudinal observation of disease progression in model organisms. Pioneered by the lab of Richard Morimoto (Morley et al., 2002; Gidalevitz et al., 2006), such sensors are usually comprised of conformationally unstable client proteins that require chaperone assistance for maintaining their native conformation. In proteotoxic stress conditions when the cellular folding capacity is consumed by other clients, the sensors misfold and aggregate. The occurrence and extent of misfolding, which can be detected by different readouts, serve as an indication of proteostasis impairment.

#### Genetically Encoded Sensors Based on Endogenous Proteins

A set of studies in *Caenorhabditis elegans (C. elegans)* models made use of conformationally destabilized endogenous proteins to detect global imbalance in protein quality control (Gidalevitz et al., 2006; Ben-Zvi et al., 2009; van Oosten-Hawle et al., 2013; Miles and van Oosten-Hawle, 2020). These endogenous proteins contain temperature-sensitive (ts) point mutations known to cause specific phenotypes at restrictive (elevated), but not permissive (control) temperatures. In proteotoxic conditions due to aging or co-expression of aggregating proteins, misfolding of the destabilized ts proteins occurs already at permissive temperature, leading to the exposure of their specific mutant phenotypes. A whole panel of ts sensors have been generated based on proteins enriched in different tissues, including muscle, neuronal, intestinal, and hypodermal cells. Among the neuronal sensors are ts versions of dynamin (Figure 2B), ras, gas-1, and acetylcholine receptor. Depending on the type of ts-protein, various readouts can be used to analyze its loss of function at the organismal level, such as movement coordination, paralysis, and lethality, as well as the sensitivity of the worms to different stress conditions. At the cellular level, the misfolded destabilized proteins mislocalize, form aggregate-like structures, and show altered sensitivity to proteolysis, indicating a disturbance in the cellular folding environment (Gidalevitz et al., 2006; Ben-Zvi et al., 2009).

Destabilized endogenous proteins have enabled several seminal discoveries on proteostasis in *C. elegans* models (Gidalevitz et al., 2006; Ben-Zvi et al., 2009; van Oosten-Hawle et al., 2013). The advantage of these sensors is their biological relevance within the cells under study. However, mutated endogenous proteins also have notable shortcomings when used as proteostasis sensors, as tissue-specific phenotypes of different proteins are difficult to compare. Another major concern is the potential loss-of-function effects resulting from the misfolding and aggregation of endogenous proteins. Several unrelated destabilized proteins can be used to ensure that the findings are not only valid for one specific client and likely do not result from its loss-of-function (Gidalevitz et al., 2006; Ben-Zvi et al., 2009), however, such studies would not be feasible in more complex organisms.

#### Genetically Encoded Sensors Based on Ectopic Proteins

Sensors based on ectopically expressed proteins lack an endogenous function, are therefore more inert, and cause less interference with cellular physiology. Such bioorthogonal sensors include different versions of luciferase, an enzyme that catalyzes a light-emitting bioluminescence reaction (Rokney et al., 2009; Winkler et al., 2010; Gupta et al., 2011; Donnelly et al., 2014; Frottin et al., 2019). In conditions of impaired proteostasis, these proteins form aggregates, visible as fluorescent foci if the sensor is fused to a fluorescent protein. In addition, luciferase offers a second way of analyzing folding efficiency, as it displays reduced enzymatic activity when not folded properly. This can be detected by measuring bioluminescence emitted in a luciferase assay, providing a reliable quantitative readout with a large dynamic range. One of such sensors made up of the luciferase from the firefly *Photinus pyralis* fused to an enhanced green fluorescent protein (Fluc-EGFP, Figure 2C), is available as a series of progressively destabilized mutants (Gupta et al., 2011), which broaden the spectrum of proteostasis capacity states that can be probed. As the pool of Fluc-EGFP is not entirely folded in mammalian cells at baseline conditions, the state of improved proteostasis, e.g., as a result of activated stress responses, can also be detected by an increase in luciferase activity. The functionality of this sensor has been proven not only in cell lines and in *C. elegans*, but also in primary murine neurons. Moreover, a transgenic mouse with Fluc-EGFP expression in the nervous system has been generated, which gives a unique opportunity to investigate *in vivo* proteostasis changes in normal aging and in mouse disease models (Blumenstock et al., 2021). Thus, the sensor revealed proteostasis impairments in a tauopathy model at an early stage, preceding neuronal cell death and behavioral symptoms. At the single-cell level, a clear reaction of the sensor was observed in tau-expressing cells even in the absence of tau neurofibrillary tangles. These findings point to proteostasis alterations as an early hallmark of neurodegeneration *in vivo*. Of note, while the bioluminescence readout showed very high sensitivity in cell lines, it proved less sensitive in bulk brain tissue of proteinopathy model mice (Gupta et al., 2011; Blumenstock et al., 2021), possibly due to the heterogeneity of neuronal cell types that differ in their reactions to misfolding. This underlines the importance of single-cell sensor readouts in order to capture the complexity of cell types *in vivo*.

Some sensors are designed to interact with a fluorogenic small molecule probe, forming a sensor-probe conjugate. One of such reporters is AgHalo, an unstable, aggregation-prone variant of the HaloTag protein (Los et al., 2008; Liu et al., 2017). The conjugate of AgHalo with its small molecule ligand is non-fluorescent in the folded state but shows a strong increase in fluorescence in a misfolded or aggregated state (Figure 2D). With an improved version of the fluorogenic probe, this reporter was shown to be highly sensitive even to early stages of protein misfolding under mild proteotoxic stress (Fares et al., 2018). In addition, possible applications of the AgHalo sensor are broadened by the availability of multicolor probes. Thus, a combination of probes can be used to simultaneously label the folded and misfolded sensor pools within the same cell and observe their dynamics in real time (Liu et al., 2018). Another sensor that works in complex with a fluorogenic small molecule is the *de novo* designed enzyme retroaldolase with destabilizing mutations. In this case, the small molecule probe becomes fluorescent upon binding to the sensor, and proteostasis disturbance is detected by a redistribution of fluorescence in a cell from diffuse to granular (Liu et al., 2015). Small molecule-regulated sensors enable precise temporal measurements of proteostasis capacity alterations, including pulse-chase approaches, as the contribution of newly synthesized sensor to the readout is excluded after the unbound probe has been washed out. The limitation of these sensors is that longitudinal studies in living organisms would require repeated delivery of the fluorogenic probe. Up to date, this type of sensor has only been used in bacteria and in mammalian cell lines.

A further group of genetic sensors takes advantage of fluorescence resonance energy transfer (FRET) between a pair of fluorophores fused to the N- and C-termini of an unstable protein to visualize the folding efficiency. One such sensor comprises the prokaryotic protein barnase attached to a cyan (mTFP1) and yellow (Venus) FRET pair (Wood et al., 2018). The FRET signal is low in the unfolded state of the sensor, medium in the folded state, and high in the aggregated state (Figure 2E). With the help of mathematical modeling, the proteostasis capacity can be quantified based on the proportion of cells in different FRET intensity states measured by flow cytometry. Like Fluc-EGFP, the barnase sensor is able to detect not only impairments but also improvements in folding efficiency. In addition, a range of progressively destabilized barnase mutants are also available (Wood et al., 2018). An important advantage of this sensor is that it provides a precise quantitative measure of the cellular folding capacity under different experimental conditions. However, it should be noted that the quantitative FRET readout of the sensor, which makes use of flow cytometry, would be challenging *in vivo*, as it requires isolation and dissociation of the cells of interest. The less quantitative evaluation of visible sensor aggregates remains feasible also in a living organism. Another FRET-based sensor consists of the mutated phosphoglycerate kinase (PGK) protein with a green/red fluorophore pair (Ebbinghaus et al., 2010). It was developed for monitoring protein folding in cells upon brief temperature jumps but has not yet been validated in the context of disease-related proteostasis impairments. In general, FRET-dependent sensors have so far only been tested in non-neuronal cell lines, and the utility of these sensors in neurons remains to be explored. When using transient transfection to express sensors based on ectopic proteins, it should be kept in mind that the poor control of the sensor expression levels might lead to a high variability of the results.

All genetically encoded sensors have the important advantage that they can be targeted to specific cell populations by using different promoters. This opens the unique possibility to dissect the relations between proteostasis of different tissues of an organism (van Oosten-Hawle et al., 2013) and potentially of different cell types within a tissue. Such applications are particularly relevant in a complex organ like the brain, containing a great variety of neuronal and glial cell types.

On the other hand, a common consideration for all genetically encoded sensors is that conformationally unstable proteins might themselves to some extent trigger stress responses and enhance adverse phenotypes in proteinopathy models (Gidalevitz et al., 2006; Gupta et al., 2011). Therefore, care should be taken when using destabilized proteins as sensors, and their expression should be kept low to avoid occupying a large fraction of the proteostasis machinery with the sensor itself. To ameliorate this limitation, inducible genetic strategies would be preferable, as they allow tighter temporal control of expression and minimize undesired stress responses and chronic adaptations of the protein quality control system. However, such strategies are more complex and require the expression of multiple constructs, limiting their use in animal models.

## Discussion and Future Directions

The available toolbox of proteostasis biosensors has been quickly expanding during the last decade. For small molecule sensors, major recent improvements include increased sensitivity, stability, and spectral properties. Genetically encoded sensors have become more biologically inert, and their readouts more quantitative. It should be kept in mind that different sensors, albeit intended to report on the global state of the proteostasis system rather than its specific branches, might be biased towards certain parts of the protein quality control machinery or certain client proteins, and might not be representative of the state of the entire proteome. The comparability of different sensors has not been systematically explored and remains to be clarified in future experiments. A combination of sensors is therefore desirable for a more comprehensive assessment of a condition under study.

Cellular compartments and organelles such as the nucleus, mitochondria, and endoplasmic reticulum have evolved distinct proteostasis pathways, and recent studies have highlighted the impact of these organelle-specific mechanisms on cellular proteostasis (Walter and Ron, 2011; Kirstein et al., 2015; Miller et al., 2015; Frakes and Dillin, 2017; Shpilka and Haynes, 2018; Frottin et al., 2019; Moehle et al., 2019). Some of the existing proteostasis sensors have already been targeted to different cellular compartments (Winkler et al., 2010; Frottin et al., 2019; Blumenstock et al., 2021; Melo et al., 2021; Raeburn et al., 2021). A set of spectrally distinct, compartment-specific sensors that could be combined with each other would be a helpful resource for elucidating intra-compartmental crosstalk.

While proteostasis sensors have proven very useful in cell culture and *C. elegans* models, investigating neuronal proteostasis in higher organisms has for a long time remained a challenge. The generation of proteostasis reporter mice (Blumenstock et al., 2021) opens exciting new avenues for fundamental insights into *in vivo* mechanisms of protein misfolding. Thus, transgenic Fluc-EGFP mice will allow longitudinal investigations of the dynamics of proteostasis impairments during disease progression at the single-cell level with modern imaging methods such as *in vivo* two-photon microscopy. In addition, thanks to the recent advances in microfluidics, single-cell RNA-sequencing, and proteomic methods, linking the *in vivo* folding efficiency of single neurons to their molecular profiles is now within reach. Comparison of the proteostasis capacity of different cell types might enable important discoveries about neuronal vulnerability to disease.

In addition to elucidating the basic mechanisms of aging and disease, proteostasis sensors hold great promise for more translational applications, particularly as biomarkers of protein misfolding disorders including neurodegeneration. Proteostasis disturbances are among the earliest hallmarks of these diseases, therefore proteostasis biosensors could be used for early diagnostics or even prophylactic screening to identify individuals at risk. Longitudinal monitoring of proteostasis alterations in biosamples from patients could furthermore provide an efficacy measure for therapeutic interventions. The use of proteostasis sensors in mammalian models is an important milestone for these exciting future clinical applications.

## Author Contributions

ID conceived and wrote the manuscript.

## Conflict of Interest

The author declares that the research was conducted in the absence of any commercial or financial relationships that could be construed as a potential conflict of interest. The handling editor IS declared a shared affiliation with one of the authors ID at time of review.

## Publisher’s Note

All claims expressed in this article are solely those of the authors and do not necessarily represent those of their affiliated organizations, or those of the publisher, the editors and the reviewers. Any product that may be evaluated in this article, or claim that may be made by its manufacturer, is not guaranteed or endorsed by the publisher.
